# Effect of immersive visualization technologies on cognitive load, motivation, usability, and embodiment

**DOI:** 10.1007/s10055-021-00565-8

**Published:** 2021-08-16

**Authors:** N. Wenk, J. Penalver-Andres, K. A. Buetler, T. Nef, R. M. Müri, L. Marchal-Crespo

**Affiliations:** 1 Motor Learning and Neurorehabilitation Laboratory, ARTORG Center for Biomedical Engineering Research, University of Bern, Bern, Switzerland; 2 Gerontechnology & Rehabilitation, ARTORG Center for Biomedical Engineering Research, University of Bern, Bern, Switzerland; 3 Department of Neurology, University Neurorehabilitation, University Hospital Bern (Inselspital), University of Bern, Bern, Switzerland; 4 Department of Cognitive Robotics, Delft University of Technology, Delft, The Netherlands

**Keywords:** Immersive Virtual Reality, Augmented Reality, Cognitive Load, Motivation, Usability, Embodiment

## Abstract

Virtual reality (VR) is a promising tool to promote motor (re)learning in healthy users and brain-injured patients. However, in current VR-based motor training, movements of the users performed in a three-dimensional space are usually visualized on computer screens, televisions, or projection systems, which lack depth cues (2D screen), and thus, display information using only monocular depth cues. The reduced depth cues and the visuospatial transformation from the movements performed in a three-dimensional space to their two-dimensional indirect visualization on the 2D screen may add cognitive load, reducing VR usability, especially in users suffering from cognitive impairments. These 2D screens might further reduce the learning outcomes if they limit users’ motivation and embodiment, factors previously associated with better motor performance. The goal of this study was to evaluate the potential benefits of more immersive technologies using head-mounted displays (HMDs). As a first step towards potential clinical implementation, we ran an experiment with 20 healthy participants who simultaneously performed a 3D motor reaching and a cognitive counting task using: (1) (immersive) VR (IVR) HMD, (2) augmented reality (AR) HMD, and (3) computer screen (2D screen). In a previous analysis, we reported improved movement quality when movements were visualized with IVR than with a 2D screen. Here, we present results from the analysis of questionnaires to evaluate whether the visualization technology impacted users’ cognitive load, motivation, technology usability, and embodiment. Reports on cognitive load did not differ across visualization technologies. However, IVR was more motivating and usable than AR and the 2D screen. Both IVR and AR rea ched higher embodiment level than the 2D screen. Our results support our previous finding that IVR HMDs seem to be more suitable than the common 2D screens employed in VR-based therapy when training 3D movements. For AR, it is still unknown whether the absence of benefit over the 2D screen is due to the visualization technology per se or to technical limitations specific to the device.

## Introduction

### VR for motor training

Virtual reality (VR) has been proposed as a promising tool to support motor (re)learning in healthy (Levac et al. 2019) and neurologic patients (e.g., after stroke) (Gobron et al. 2015; Perez-Marcos et al. 2018). During VR-based movement training, users engage in meaningful goal/task-oriented exercises while visualizing their movements reproduced in the virtual environment (VE). In the context of rehabilitation technology, VR has been defined as “an advanced form of human–computer interface that allows the user to *interact* with and become *immersed* in a computer-generated environment in a naturalistic fashion” (Schultheis and Rizzo 2001). A “computer-generated” environment can also be called a “virtual” environment (VE), which was described by Blascovich as “an organization of sensory information that leads to perceptions of a synthetic environment as non-synthetic” (Blascovich 2002). The immersion in this VE is defined as the extent to which the computer systems are extensive—relates to the number of sensory systems they target—, surrounding—i.e., the capability to create stimuli from multiple directions—inclusive—i.e., the capability to hide stimuli from the real world—, vivid—relates to the variety and the richness of the generated stimuli—, and matching—the real users’ proprioceptive feedback (Slater et al. 1995).

Although VR-based interventions have shown promising results on movement training (Marchal-Crespo et al. 2013; Sigrist et al. 2013), balance and gait training (Keshner and Lamontagne 2021), and upper-limb function recovery after stroke (Domínguez-Téllez et al. 2020; Mekbib et al. 2020), especially in increasing users’ motivation, enjoyment, and engagement (Bernardoni et al. 2019; Maclean et al. 2000; Maclean and Pound 2000; Putrino et al. 2017), their benefits may still be limited due to the currently employed displays. The most common displays employed during movement training are standard computer screens, televisions, or wall projection systems (Laver et al. 2017; Mekbib et al. 2020). These visualization technologies (referred to as “2D screens” in this paper) render the VE on a two-dimensional (2D) surface that only allows visualizing a third dimension (depth) with monocular cues (Riener and Harders 2012). Although certainly useful, these monocular depth cues lack stereopsis, potentially hampering the naturalistic perception of depth, and thus, hindering the execution and visualization of functional three-dimensional (3D) movements.

Other than depth perception, rendering the VE on 2D screens results in further limitations. First, users need to perform an extra visuospatial transformation from the arm movement space’s coordinate system to the 2D screen space’s coordinates. Second, the eye-hand coordination (Rizzo et al. 2017) is uncoupled (Mousavi Hondori et al. 2016). Third, the virtual representation of the user’s limb in the VE is generally simplistic—e.g., in the form of a cursor (Ferreira dos Santos et al. 2016). Further, the immersion—identified as an advantage in VR-based interventions in healthcare—reaches a relatively low level with 2D screens (Rose et al. 2018). Therefore, current standard VR-based training paradigms involve movements and interactions with virtual objects that significantly differ from those in the real world. For example, the lack of direct naturalistic interaction and low immersion could increase the cognitive effort during training, negatively impacting motor (re)learning. Current VR-based motor training may, therefore, not only limit the transfer of acquired skills into activities of daily living but also limit users’ inclusion and adherence to VR-based motor training programs.

The implementation of new commercially available low-cost head-mounted displays (HMDs) in movement training is promising, as they allow highly naturalistic interaction and immersion with and in the VE—e.g., users can visualize their limbs’ movements in real-time as avatars from a first-person perspective (Charles et al. 2020; Perez-Marcos et al. 2018; Wenk et al. 2020). The HMDs can be classified into two main categories: (1) (immersive) VR HMDs that place the user in a computer-generated environment, and (2) augmented reality (AR) and mixed reality HMDs that render the virtual elements on top of the real environment. With VR HMDs, the immersion in the VE is usually considered higher than with a 2D computer screen (Rose et al. 2018), thus, HMD-based VR is often referred to as (more) immersive VR (IVR) (Bailey and Bailenson 2017; George et al. 2018; Slater et al. 2010). Current off-the-shelf HMDs incorporate a stereoscopic display and head-tracking capabilities, providing** close to natural depth perception**—i.e., reproducing stereopsis and motion parallax (Riener and Harders 2012; Zhan et al. 2020). The HMDs can also provide a **highly realistic movement visualization** by mapping the users’ movements on a realistic virtual avatar instead of an abstract representation.

### HMDs: Current applications

#### General applications of HMDs

HMDs are well-known in the media industry—e.g., to enhance the users’ level of immersion in videos or to provide users with a more naturalistic interaction with video games (Mütterlein and Hess 2017). In recent years, the use of HMDs has emerged in other relevant fields, such as social learning spaces—e.g., classrooms or museums; Scavarelli et al. 2021—and other industrial applications such as architecture, engineering, and construction (Alizadehsalehi et al. 2021, 2020; Alizadehsalehi and Yitmen 2021). Important clinical applications of immersive VR HMDs include the provision of VR therapy for the non-pharmacological treatment of pain (Pourmand et al. 2018; Theingi et al. 2021), and the treatment of mental disorders in a safe and controllable setting—e.g., eating disorders (Matamala-Gomez et al. 2021), anxiety disorders such as phobias (Boeldt et al. 2019), and post-traumatic stress disorders (Oing and Prescott 2018). HMDs have also been successfully employed to reduce patients’ cognitive decline (Gerber et al. 2019; Sokolov et al. 2020).

A remarkable application of VR and AR HMDs is to increase safety and accuracy in the field of **surgery** while minimizing complications and costs (Longo et al. 2021). During surgical procedures, AR HMDs might be employed to display relevant and assistive information in a congruent 3D space on top of the operating table (Andrews et al. 2019; Longo et al. 2021). The IVR HMDs are used during preoperative planning and simulation training to practice specific motor skills in a safe environment (Longo et al. 2021). Several principles of motor learning have been gained from IVR-based surgery experiments (Maier et al. 2019). For example, during surgery training, a strong interest lies in trainees visualizing their movements as they would do in real settings (i.e., task-specific training) to maximize the transfer of the learned skills into real life. Some HMDs are exploited in these training environments as they allow the reproduction of depth cues that are meaningful for the surgeons during the actual procedures (Longo et al. 2021; Lungu et al. 2021).

#### HMDs in neurorehabilitation

HMDs have also raised enthusiasm in neurorehabilitation specialists. The use of HMDs was rated as having a strong potential for rehabilitation by health specialists (Gobron et al. 2015), and was positively evaluated (Elor et al. 2018), and reached high-acceptance among people of different ages and neurological conditions without inducing serious side effects—i.e., motion-sickness (Christou et al. 2018; Kourtesis et al. 2019; Lee et al. 2020; Weber et al. 2019). The new generation of commercially available HMD (from 2013 and after) coupled with ergonomic interactions seems to be a promising approach to deliver VR-based movement training in a naturalistic and immersive manner with high user acceptance (Kourtesis et al. 2019).

HMDs have been employed in different domains of stroke rehabilitation (see Table 1 for an overview), such as cognitive training (Gamito et al. 2017) and visual neglect assessment (Knobel et al. 2020). However, literature on HMD interventions for motor training is still scarce (Riva et al. 2020; Rose et al. 2018). Only a few studies have investigated the use of HMDs for motor neurorehabilitation within the last ten years. Examples include the combination of HMDs with rehabilitation robotic devices for telerehabilitation (Perez-Marcos et al. 2012), balance training (Jung et al. 2012), gait rehabilitation (Lee et al. 2014), and upper-limb rehabilitation (Lee et al. 2020).

**Table 1:** List of studies that investigate the use of HMDs for post-stroke neurorehabilitation

Authors	Display (HMD)	Input	Participants	Task	Avatar	Outcomes
(Perez-Marcos et al. 2012)	NVIS SX 111	Rehabilitation robot (GRAB)	–	Path following	Avatar from 1st-person perspective	Proof of concept
(Jung et al. 2012)	Mybud Accupix	–	21 stroke patients	Passive walk immersion	Not specified	Balance was higher when HMD used compared to standard treadmill training
(Lee et al. 2014)	i-visor fx601 HMD + camera for video see-through AR	No movement acquisition	21 stroke patients	Lower limbs movement reproduction (imitating a template)	Real body visible	The addition of AR-based postural training to standard therapy improved motor performance
(Gobron et al. 2015)	Oculus Rift DK 2	Rehabilitation robot (LHS)	33 health specialists	4 games for the lower limb (2 using HMDs)	No avatar in immersive games	HMDs rated as convincing
(Gamito et al. 2017)	eMagin Z800	Not specified	20 stroke patients	Several games for cognitive training	No avatar	The developed IVR tasks led to improvements in attention and memory function
(Christou et al. 2018)	HTC Vive	HTC Vive tracker on a stick	11 chronic stroke patients	Path following	Floating tool	HMD well-tolerated, without fatigue or nausea
(Elor et al. 2018)	HTV Vive	HTC Vive controllers	9 stroke patients	Reaching task in 3D space	No avatar	Feasibility study: The HMD system was rated positively
(Lee et al. 2020)	HTC Vive	HTC Vive controller	12 stroke patients	5 mini-games involving movements in 3D space	Floating forearms	No adverse effect, high satisfaction, and functional improvements. No control group
(Weber et al. 2019)	Oculus Rift	Oculus Rift controller	10 chronic stroke patients	Immersive mirror therapy	Avatar from 1st-person perspective	IVR well-tolerated, no adverse events, and trend for motor improvements. No control group
(Knobel et al. 2020)	HTC Vive	HTC Vive controller	15 stroke patients	3D visual search	Floating controller	High usability and acceptance

### Potential benefits of HMDs for motor training

The use of more immersive VR in motor training settings has been encouraged by recent reviews (Keshner and Lamontagne 2021; Levac et al. 2019; Mekbib et al. 2020). In an IVR system with an avatar visualized from a first-person perspective, **visuospatial transformations** from the performed movement to its virtual representation are **minimized** and the **natural eye-hand coordination is preserved**. These aspects might reduce the user’s cognitive load, accelerating motor learning—especially during early learning phases (Schweighofer et al. 2018)—and allow patients with severe cognitive impairments to enroll in less-demanding VR-based motor training.

Further, the rendering of realistic virtual representations of the users’ limbs in a highly immersive HMD may enhance their **embodiment** over the avatar. Embodiment results from the integration of multimodal sensory information (i.e., somatosensory and visual) in the brain (Botvinick and Cohen 1998; Ehrsson et al. 2004). Numerous studies have shown that body ownership can be experimentally induced over virtual limbs in healthy subjects (Kilteni et al. 2012a; Slater et al. 2008) and stroke patients (Borrego et al. 2019). Importantly, neuroimaging studies have shown that brain areas linked with embodiment overlap with those involved in motor control (Ehrsson et al. 2005; Wise 1985; Zeller et al. 2016). Thus, increasing virtual embodiment through specialized displays—such as HMDs (Spanlang et al. 2014)—might be an effective tool to promote brain plasticity and improve motor (re)learning (Grechuta et al. 2017; Odermatt et al. 2021; Shibuya et al. 2018).

**Motivation**, subjectively assessed through well-established questionnaires (i.e., the “Intrinsic Motivation Inventory”; IMI; Reynolds 2007), during training is crucial to enhance motor learning (Wulf and Lewthwaite 2016) and recover function post-stroke (Maclean et al. 2000; Maclean and Pound 2000; Putrino et al. 2017). Thus, VR-based interventions generally aim at enhancing users’ motivation, enjoyment, and engagement during movement training by integrating meaningful, versatile, and individualized motor tasks (Rohrbach et al. 2019). However, to the best of our knowledge, no previous studies have measured the impact of different visualization displays on users’ motivation during a reaching task.

An important factor, usually not well addressed during the development of rehabilitation technology (Koumpouros 2016), is the **usability** of a novel system. System usability, subjectively quantified through well-established questionnaires (i.e., the “System Usability Scale”; SUS; Brooke 1996), refers to the capability to allow the user to effectively achieve the intended objective (effectiveness) with minimal effort (efficiency) and high satisfaction (Frøkjær et al. 2000). A high usability of a novel technology will, therefore, substantially determine patients’ adherence and therapists’ acceptance to technology-based neurorehabilitation programs. Whereas in other fields—e.g., website or user experience (UX) design—questionnaires are occasionally employed, literature presenting systematic experimental evidence in usability as a function of the technology—and its potential effect on neurorehabilitation—is scarce (Koumpouros 2016).

Together, the more naturalistic interaction and immersion with and in the VE provided by novel off-the-shelf HMDs (Bailey and Bailenson 2017) could have a positive impact on the users’ cognitive load, motivation, technology’s usability, and embodiment. When applied to VR-based movement training, HMDs could potentially improve the overall learning outcomes. However, despite their potential for neurorehabilitation, to date, HMDs are not extensively used in clinical settings—i.e., less than 6% in the latest Cochrane review (Laver et al. 2017) or not represented at all in more recent reviews (Mekbib et al. 2020). One reason might be the limited studies available that tried to quantify cognitive and psychological benefits associated with the use of HMDs, making it difficult to estimate their full clinical potential.

### Comparing different visualization technologies: current evidence of their influence on motor performance and user’s acceptance

Several efforts have been made to compare the benefits associated with training using different VR or AR visualization technologies. For example, in the construction industry, a recent study compared the effort (in terms of hours and cost) needed to create replicates of spacecraft habitats using either IVR HMD, AR HMD or a physical reproduction (Banerjee et al. 2021). While the effort was higher in any HMD compared to the physical reproduction, the authors concluded that IVR HMD might lower the costs associated with reproducing physical components.

Perhaps more relevant for HMD-based motor training of upper-limbs are the studies that compared the effect of different visualization technologies on user’s performance and technology acceptance (see Table 2 for an overview of current evidence). Regarding 3D motor tasks, a study reported no significant differences between 2D screens and IVR HMDs in motor performance, acceptance or cybersickness in stroke patients (Dias et al. 2019). However, patients preferred training with the HMD. In an earlier study, a decrease in movement performance was associated with IVR HMD compared to a projected 2D screen in both healthy participants and stroke patients (Subramanian and Levin 2011). However, in this study, participants performed an arm-pointing task on a two-dimensional surface (matching the projected 2D screen’s horizontal and vertical axes), minimizing the potential advantages of the additional depth cues provided by HMDs over 2D screens. Focusing on depth perception, the motor performance of healthy participants during reaching movements was evaluated to drive practical suggestions for the design of VR-based training therapy (Gerig et al. 2018). Authors compared an off-the-shelf IVR HMD (HTC Vive, HTC, Taiwan & Valve, USA) with differently reproduced monocular depth cues (e.g., aerial and linear perspective, shadows, and occlusion) in a 2D computer screen. They found that the IVR HMD led to better movement performance compared to the 2D screen independently of the amount of recreated depth cues. Similarly, another study found that disabling the stereopsis in an HTC Vive HMD hampered healthy participants’ motor performance while performing a path-following task (Christou et al. 2018).

**Table 2:** List of studies comparing different VR or AR visualization technologies

Authors	Displays	Input	Participants	Task	Avatar	Outcomes
(Subramanian and Levin 2011)	Kaiser XL-50 HMD (IVR) vs. 2D projected screen	Optotrack Certus Motion Capture System	10 healthy participants + 20 stroke patients	Pointing	No avatar	Better motor performance with 2D screen
(Mousavi Hondori et al. 2016)	Projected AR vs. 2D computer screen	Vision-based system	18 chronic stroke patients	Reaching on a 2D plane	No avatar (real arm seen in AR)	Better motor performance in AR, linked to a decrease in cognitive load, but not measured
(Christou et al. 2018)	HTC Vive HMD (IVR) with stereopsis vs. without	HTC Vive tracker on a stick	18 healthy participants	Path following	Floating tool	Stereopsis increases motor performance
(Gerig et al. 2018)	HTC Vive HMD (IVR) vs. 2D computer screen	HTC Vive controller	10 healthy participants	Reaching with different recreated depth cues	Floating controller or symbolic representation	Better task performance with HMD, no matter the recreated depth cues
(Krichenbauer et al. 2018)	Oculus Rift DK2 HMD (IVR) vs. ovrVision on HMD (AR)	Mouse vs. 6 DoF tracked controller	24 healthy participants	Object selection and transformation task with 9 DoF	No avatar in IVR and real body visible in AR	Faster completion time in AR, but no difference in reported comfort
(Chicchi Giglioli et al. 2019)	HTC Vive HMD (IVR) vs. HoloLens (AR)	HTC Vive controller vs. HoloLens’ hand tracking	36 healthy participants	Cooking simulation with two hands	Floating controllers in IVR	Higher levels of presence and smaller completion time in IVR, linked to differences in input techniques
(Dias et al. 2019)	Oculus Rift DK2 HMD (IVR) vs. 2D computer screen	Leap motion	12 stroke patients	3 mini-games involving movement in 3D space	Floating hand in unnatural blue color	No significant differences in neither motor performance nor acceptance, no cybersickness, but a preference for IVR
(Wenk et al. 2019)	HTC Vive HMD (IVR) vs. Meta 2 HMD (AR) vs. 2D computer screen	HTC Vive controller	20 healthy participants	Reaching in a 2D space and counting (cognitive) task	Avatar from 1st-person perspective and real body visible in AR	Better movement performance in IVR than with the 2D screen. Multidimensional movements deteriorate only with the 2D screen. No differences in cognitive task

Only a few studies have evaluated the potential benefits associated with the use of AR in motor (re)learning. In a cooking-like task, IVR HMD was found to outperform AR HMD in terms of task performance and sense of presence (Chicchi Giglioli et al. 2019). However, the authors hypothesized that the advantage of IVR over AR HMDs might be due to each HMD-specific input device—i.e., a controller in IVR vs. the hand in AR. Conversely, in (Krichenbauer et al. 2018), the authors found that training with an AR HMD resulted in better movement performance during object manipulation when compared to training with an IVR HMD—without avatar—in healthy participants. This might underline the positive impact of visualizing the movements performed by our body on motor performance. A simplistic movement visualization was also identified as a potential reason behind poor movement quality in a 1D reaching task in VR compared to movements in real life (Robert and Levin 2018).

In the field of stroke rehabilitation, Mousavi Hondori and colleagues performed an experiment with 18 stroke survivors with a more comparable movement visualization between displays (Mousavi Hondori et al. 2016). The authors reported better movement performance in a 2D reaching task (performed by moving the hand on a table) when using a 2D surface projection (i.e., AR without an HMD) vs. a 2D computer screen. However, the 2D computer screen was located on a vertical plane, whereas the 2D projected surface was on the same horizontal plane where the movements were performed. Therefore, the eye-hand coordination in the 2D computer screen modality was disrupted, and there was an extra visuospatial transformation between the movements performed and their visualization. The authors suggested that the lower performance related to 2D computer screen visualization might be due to an increased cognitive load associated with the extra visuospatial transformation. Unfortunately, the cognitive load was not assessed, and therefore this conclusion remains purely speculative. To date, no studies have systematically evaluated the cognitive load and other relevant subjective experiences associated with the use of different visualization technologies.

### Aim of the present study

In this study, we aimed at evaluating the effect of performing a dual motor-cognitive task (i.e., reaching fruits in 3D space and counting different fruit types) with the following three different visualization technologies on participants’ self-reported cognitive load, motivation, technologies’ usability, and embodiment: (1) IVR HMD (HTC Vive, HTC, Taiwan & Valve, USA), (2) AR HMD (Meta 2, Meta View, USA), and (3) 2D computer screen (thus providing only monocular depth cues). In a first analysis focusing on the impact of visualization technologies on motor and cognitive task performance—published as a conference paper elsewhere (Wenk et al. 2019)—we found better movement performance with IVR than with the 2D screen. We also found tendencies suggesting a decline in movement performance with AR vs. IVR, but better movement quality compared to the 2D screen. No differences across visualization technologies were found when the score of the parallel cognitive counting task was analyzed. We hypothesized that participants might have prioritized the cognitive over the motor task, and therefore, the cognitive load imposed by the additional visuospatial transformation associated with the 2D screen might have degraded the movement performance—e.g., perhaps participants took a longer time to think about the counting value, which might have been reflected in the motor performance metrics, although they counted with the same precision. Therefore, subjective reports assessing the cognitive effort during task performance might reflect potential modulations of cognitive demands that were missed with the parallel counting task.

Our hypotheses were: (1) HMDs will reduce the subjective cognitive load compared to the 2D screen. Due to the previous inconclusive results on motor performance with AR, we expect this result to be especially visible with the IVR HMD; (2) HMDs will result in higher reported motivation compared to the 2D screen (either directly due to the differences in embodiment, immersion level, and natural interaction) or indirectly (due to the motor performance improvement observed in the previous analysis); (3) HMDs will result in a more usable system, as they reflect a more natural visualization; (4) AR would result in higher embodiment levels (as participants can look at their own limbs), followed by IVR (as an avatar was employed, respecting the body location).

To the best of our knowledge, this is the first study to compare self-reported cognitive load, motivation, technologies’ usability, and embodiment using questionnaires while performing the same therapy-inspired 3D reaching task with different visualization technologies. The ultimate goal of our research is to improve neurorehabilitation, but patients cannot be used as guinea pigs for every new technology. Therefore, we performed a first study with unimpaired participants: first, to mature the study design and implementation as much as possible, and second to provide a rapidly controlled study with sufficient statistical power, while minimizing variability in the measured variables—e.g., minimizing confounds introduced by inter-individual variability associated with stroke recovery (Prabhakaran et al. 2008)—, to rigorously analyze the training system. Insights obtained from healthy populations are of high relevance for the definition of sequential applied—but also more restricted—clinical study protocols with brain-injured patients.

## Methods

### Participants

The recruitment of participants was performed within the University of Bern via word-of-mouth. Twenty healthy participants (15 female, 5 male) without known motor or cognitive disorders, aged from 19 to 42 years old (23.65 ± 4.43) with a preference to use the right hand (Edinburgh handedness questionnaire mean score: 91 ± 19.71; Oldfield 1971) participated in the study. Participants did not receive any compensation for their participation in the study. They provided written informed consent to participate in the study. The study was approved by the local Ethics Committee (ref.: 2017–02,195) and conducted in compliance with the Declaration of Helsinki. A detailed list of participants’ genders, ages, highest education, and experiences with VR and gaming can be found in Table 3.

**Table 3:** Participants’ demographics. Experience with VR and video gaming rated from 1 (“Not at all”) to 7 (“Very much”)

Id	Gender	Age	Highest educational achievement	Experience with VR	Experience with video games	Hours spent playing video games per week in the last month
1	Male	19	Obligatory school	1	4	0.5
2	Male	26	University or equivalent	5	7	5
3	Female	23	High school	1	3	0
4	Female	23	Apprenticeship	2	2	0
5	Female	21	High school	1	2	0
6	Male	23	None	1	5	4
7	Female	22	High school	1	1	0
8	Female	22	Apprenticeship	3	2	0
9	Male	23	High school	1	7	0
10	Female	24	University or equivalent	7	2	0
11	Female	23	High school	4	5	0
12	Female	23	High school	5	6	0
13	Male	42	University or equivalent	2	5	1
14	Female	23	High school	1	2	0
15	Female	21	High school	1	1	0
16	Female	23	High school	3	1	0
17	Female	24	University or equivalent	2	2	0
18	Female	22	University or equivalent	4	3	0
19	Female	24	High school	2	1	0
20	Female	22	University or equivalent	1	1	0

### Experimental setup: visualization technologies

**Fig. 1:**
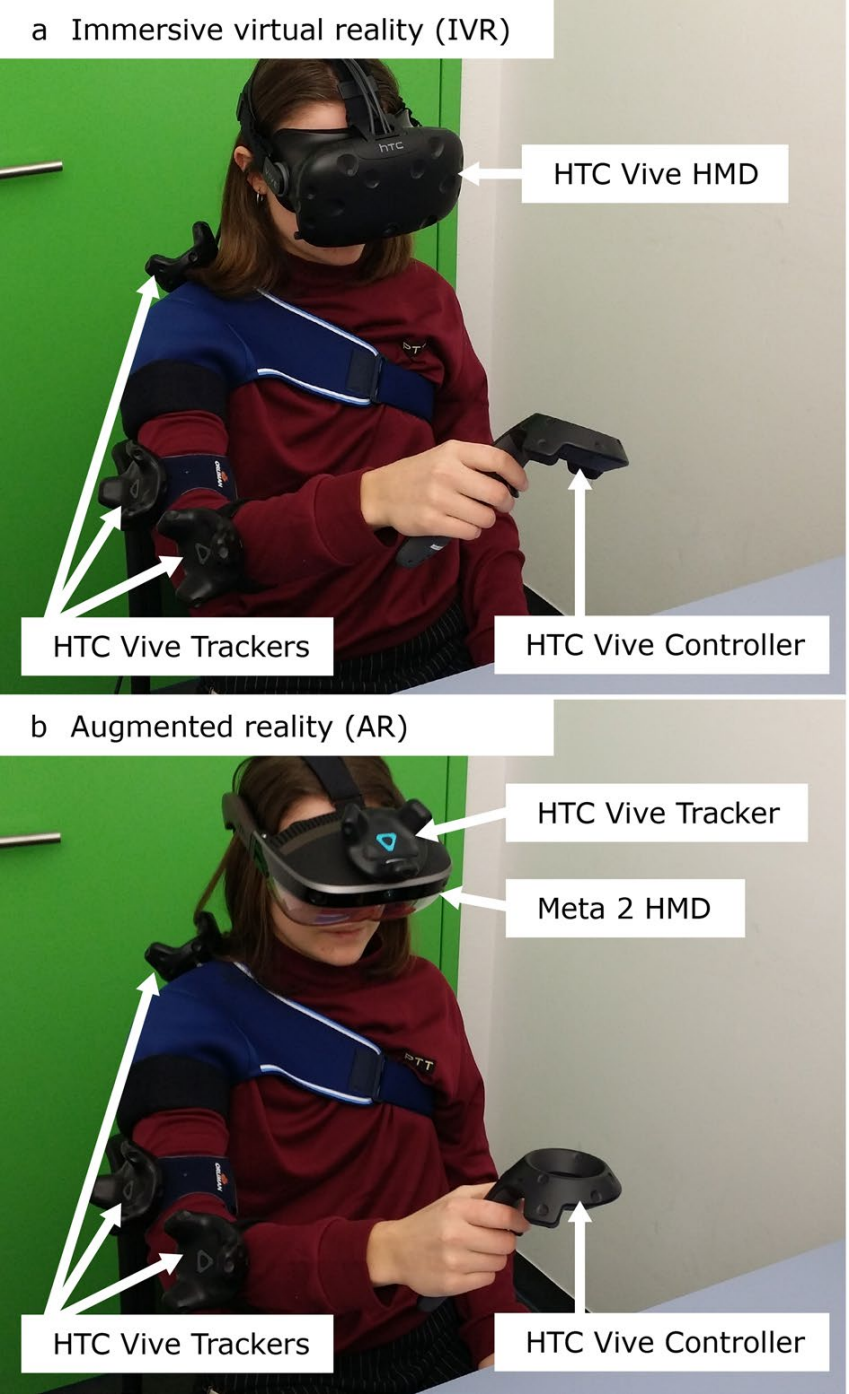
Experimental setup for the two HMD modalities. **a** Immersive virtual reality (IVR) using the HTC Vive (HTC, Taiwan & Valve, USA); **b** Augmented reality (AR) using Meta 2 (Meta View, USA)

The experiment was conducted in a room with only controllable artificial light. Participants were seated on a chair in front of a table. Three HTC Vive trackers (HTC, Taiwan & Valve, USA) were attached to the participant’s right arm and shoulder to record their movements (Fig. 1). Participants were requested to hold a tracked controller from the HTC Vive VR system (HTC, Taiwan & Valve, USA) in their right hands. The HTC Vive controller and trackers were in place in all visualization modalities and movement data were recorded using the same technical means.

The most relevant characteristics for each visualization display are summarized in Table 4. The HMD from the HTC Vive system was employed for the IVR HMD modality (Fig. 1a) as it allows easy and precise tracking of the participants’ movements using trackers (Niehorster et al. 2017). A Meta 2 HMD (Meta View, USA) was interfaced for the AR condition. The Meta 2 was, by the time we developed the experiment, the AR HMD with the largest field of view on the market and allowed us to use a relatively large task workspace. Although the Meta 2 incorporates simultaneous localization and mapping (SLAM) technology to track the user’s head and hands, we aimed at preventing that differences in head-tracking technologies would play a role in the experiment results. Disabling the Meta 2’s SLAM function and using an HTC Vive tracker attached to the Meta 2 HMD allowed us to employ the same head-tracking technology in both visualization technologies (Fig. 1b). A calibration was performed before each modality using HMDs. For the AR modality, the Meta 2 eye calibration software was employed. This calibration could last a few minutes (~ 5 min) as the researcher had to help participants to wear the HMD and guide them through a five-step calibration process. For the IVR modality, the calibration was performed by measuring the participant’s interpupillary distance and setting it in the HMD with the dedicated wheel.

**Table 4:** Characteristics of the visualization technologies

Visualization technologies	Field of view (diagonal)	Resolution (pixels)	Weight (g)
IVR–HTC Vive	145°	2160 × 1200	555
AR–Meta 2 (+ Vive tracker)	90°	2560 × 1440	420^1^ (+ 89)
2D screen–Samsung S24E560	99.33°	1600 × 900^2^	Not relevant

^1^Meta 2 HMD’s weight is provided without cables and strap

^2^The provided resolution value corresponds to the window that rendered the VE (not in full screen)

For the 2D screen modality, a Samsung S24E560 (Samsung, South Korea) computer screen was employed, as it represents the typical computer screens found in therapy settings to display information only with monocular depth cues. The screen was placed on a table approximately 80 cm away from the participant (participants could freely move their upper body) and slightly to their left side, so the arm moving within the workspace would not occlude the screen. To align the virtual camera, rendering the VE on the 2D screen with the participants’ heads, the Meta 2 HMD with the HTC Vive’s tracker was shortly worn by the participants at the start of the 2D screen modality. The researcher would remove the HMD after calibration (< 1 s) and participants performed the task by only looking at the 2D screen. The 2D screen was placed on the table also during the other modalities but was turned off during the AR modality and not visible to participants during IVR.

The experiment was developed using the game engine Unity (Unity Technologies, USA), version 2018.3.0f2. To interface with the HTC Vive’s HMD, controller, and trackers, the SteamVR (Valve Corporation, USA) plugin version 1.2.3 for Unity was employed. The Meta 2 was interfaced using the Unity SDK included in the Meta SDK2 Beta 2.7.0.38. The depth occlusion option of the Meta 2 was enabled—i.e., virtual elements were not visible when they were behind the participants’ arms. The avatar was modeled using MakeHuman v1.1.1 (MakeHuman Team, www.makehumancommunity.org) and animated using Unity’s Inverse Kinematics. The computer operated with Windows 10 Home 64 bit edition (Microsoft, USA) and ran the task within the Unity Editor. The computer possessed 32 GB of DDR3 working memory, an NVIDIA GeForce GTX 1080 Ti GPU (NVIDIA Corporation, USA), and an Intel Core i7-8700 K processor (Intel Corporation, USA).

### The dual motor-cognitive task and virtual environments

**Fig. 2:**
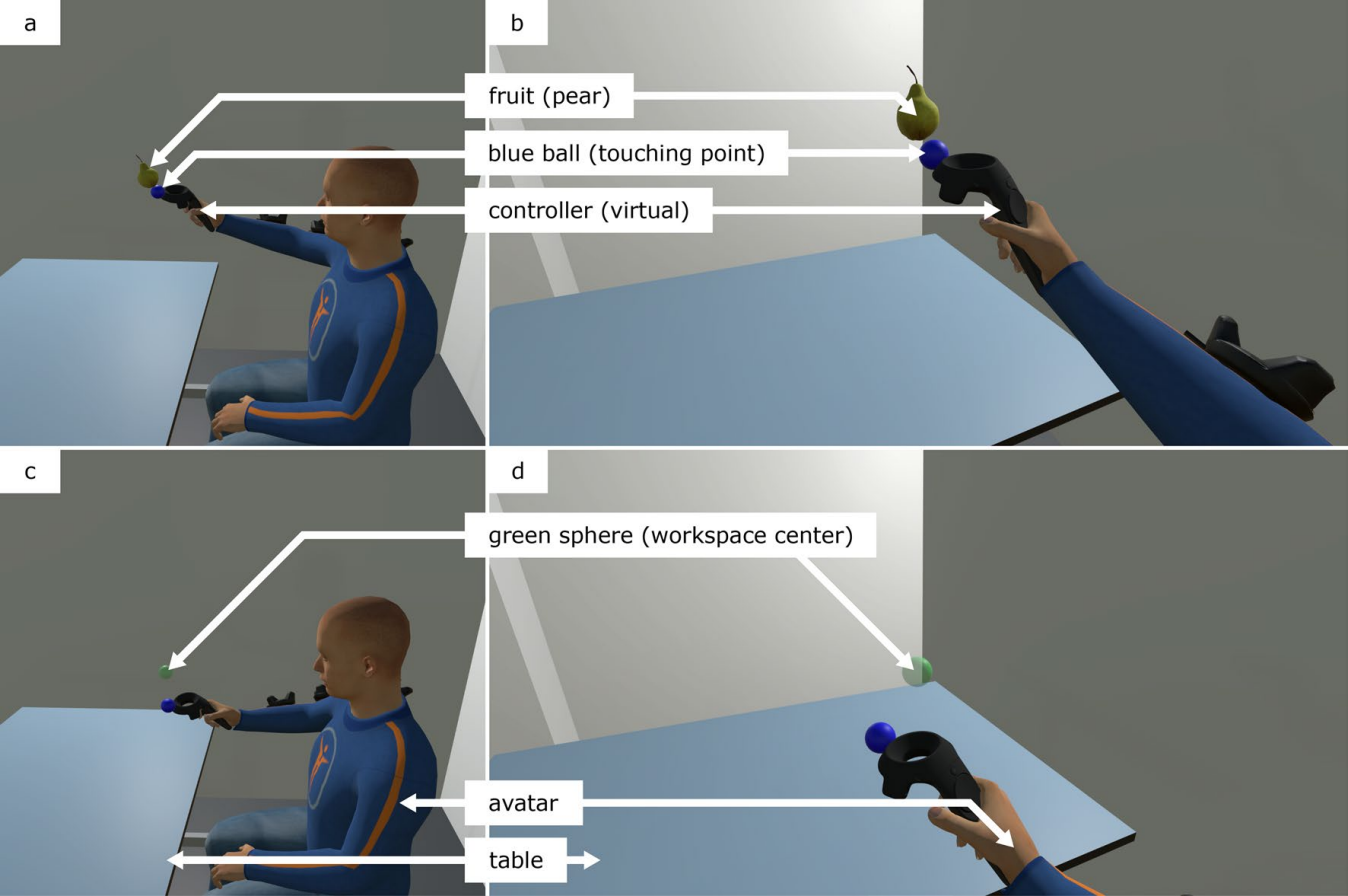
Virtual environment (VE) that includes the virtually reproduced table, walls, fruits, spheres, controller, and the avatar. **a** & **b** Reaching towards a fruit (pear); **c** & **d** Going to the workspace center; **a** & **c** Third-person perspective showing the avatar; **b** & **d** First-person perspective (real participants’ point of view). In AR, only the virtual fruits and spheres were visible to the participants

In each modality, participants performed the same dual-task visualized with different displays. The motor task consisted of sequentially reaching towards 120 fruits that appeared randomly in one of 22 possible locations. The task workspace had its center located 50 cm in front and at the same height as the right shoulder (calibrated for each participant). To reach for a fruit, participants had to “touch” the fruit with a virtual blue ball rendered at the controller’s more distal location (Fig. 2a, b). After reaching for a fruit, it disappeared and participants moved back (with the blue ball) to a green sphere that appeared at the center of the workspace (Fig. 2c, d). The predefined fruit locations required participants to move in either one (along the x-axis, y-axis, or z-axis) or several dimensions (along two or three coordinate axes) in a Cartesian coordinate system (Wenk et al. 2019). The axes of the coordinate system matched the participants’ point of view—i.e., in the 2D screen modality, the x- and y-axes correspond to the 2D screen surface. For the two-dimensional movements, only a horizontal plane (x- and z-axes) and a vertical plane facing the participant (x- and y-axis) were used. Movements along the z-axes in the 2D screen (i.e., perpendicular to the 2D screen) could not be perceived with the eyes’ vergence (as with the HMDs) but were facilitated with monocular depth cues, namely, using occlusion, shadows, and perspective-related cues. The presentation of the 120 fruits was divided into 8 blocks. For each new fruit, participants were asked to count out loud the number of previously collected fruits (within the ongoing block) of its type (orange, apple, or pear). More information on the workspace dimension, block structure, and fruit locations can be found in the conference proceeding where the task performance was analyzed (Wenk et al. 2019).

The VE was identical in the IVR and 2D screen modalities (Fig. 2). A basic reproduction of the experiment room was generated, matching the real room’s dimensions, colors, light intensity, and light location. The virtual reproduction of the furniture was reduced to the table. A fixed-size full-body humanoid avatar was employed with a fixed seated posture. The whole avatar moved (3D translation) following the HMD in IVR while it remained static during the 2D screen modality. The avatar’s spine and neck rotations were animated with inverse kinematics in IVR to match with the participants’ tracked head orientation. During the 2D screen modality, the avatar’s head and spine animation was fixed to a predefined constant orientation facing the workspace. The right arm was animated with inverse kinematics (employing the HTC Vive’s controller position and orientation) on three points (shoulder, elbow, and wrist). The avatar was rendered with the three virtual HTC Vive trackers on the arm and holding an HTC Vive controller in the hand (matching its tracked position). The VE in the AR modality consisted of only the fruits, the green ball, and the blue ball, which were lighted with the same light sources as in the other two modalities. The HTC Vive’s controller was rendered black and unlit in AR, respecting the real controller position and orientation, to occlude all game elements that were behind the controller (the Meta 2’s depth occlusion based on the embedded camera performed poorly in detecting the controller’s material).

### Study protocol

**Fig. 3:**
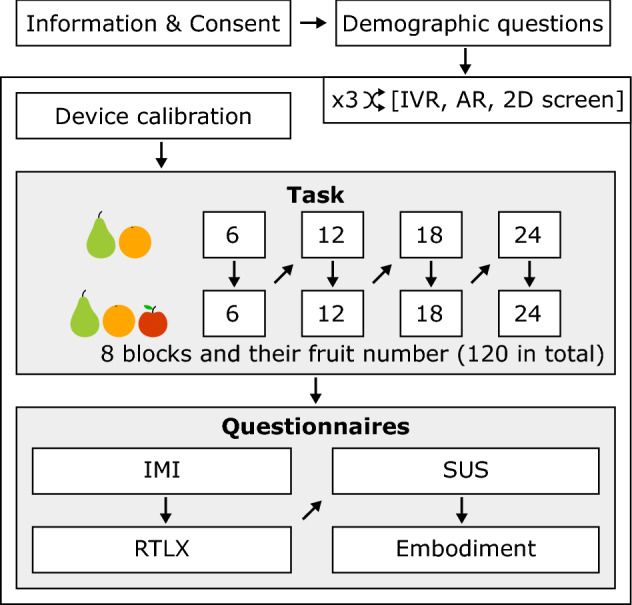
Experimental protocol. A within-subject design was performed with the three visualization modalities presented in a randomized order. The overall experiment lasted around one hour in a single session. Each modality took an average of 15 min with only 5 min of active movement

A researcher was present in the room during the whole experiment. A within-subject design was chosen to evaluate the effects of the three different visualization technologies on healthy participants’ cognitive load, motivation, technologies’ usability, and embodiment (Fig. 3). After being briefed about the experiment objectives and task details, participants answered an initial set of demographic questions. Those included questions about handedness (“Edinburgh Handedness Inventory”—EHI; Oldfield 1971), gender, birth date, education level, VR experience, gaming experience, and gaming frequency (see Table 3).

Next, participants performed the dual motor-cognitive task under the three modalities (IVR, AR, and 2D screen) in a randomized order. The order randomization resulted in nine participants starting with IVR, six with AR, and five with the 2D screen, six participants finishing with IVR, seven with AR, and seven with the 2D screen. When changing the modality, the researcher switched the display physically on the computer and performed a short calibration for each participant and display (less than a minute with IVR and the 2D screen and up to five minutes with AR). During the motor-cognitive task, the researcher was taking note of the counting values said out loud. The 120 fruits per modality were divided into eight blocks with the following fruit numbers: 6, 6, 12, 12, 18, 18, 24, and 24. Blocks 1, 3, 5, and 7 contained only oranges and pears, and blocks 2, 4, 6, and 8 contained oranges, pears, and apples (Fig. 3). After each modality test, participants answered the questionnaires related to their motivation, cognitive load, technology’s usability, and embodiment (see Sect. 2.5). Between blocks, participants were allowed to rest their arms as long as needed without removing the headset. The whole experiment had an average duration of around one hour.

### Questionnaires

After performing the dual motor-cognitive task with each modality, participants were requested to fill in questionnaires to report their subjective cognitive load, motivation, technology’s usability, and embodiment (i.e., a total of 3 times the same questionnaires). The questionnaires were filled using REDCap electronic data capture tools (Harris et al. 2009) hosted at the University of Bern. All questionnaires were translated into German.

To assess the cognitive load, the well-established “Raw Task Load Index” (RTLX; Hart 2006) questionnaire—a short version of the “Task Load Index” (Hart and Staveland 1988)—was selected. The RTLX is divided into six subjective subscales, that target *Mental Demand*, *Physical Demand*, *Temporal Demand*, *Performance*, *Effort*, and *Frustration* (Table 5).

**Table 5:** Questions from the “Raw Task Load Index” (RTLX; Hart 2006). Each question was answered using a markless slider on a scale encoded with 100 intervals without displayed numerical values

Subscale	Endpoints	Description
Mental Demand	Low/High	How much mental and perceptual activity was required (e.g., thinking, deciding, calculating, remembering, looking, searching, etc.)? Was the task easy or demanding, simple or complex, exacting or forgiving?
Physical Demand	Low/High	How much physical activity was required (e.g., pushing, pulling, turning, controlling, activating, etc.)? Was the task easy or demanding, slow or brisk, slack or strenuous, restful or laborious?
Temporal Demand	Low/High	How much time pressure did you feel due to the rate or pace at which the tasks or task elements occurred? Was the pace slow and leisurely or rapid and frantic?
Performance	Low/High^1^	How successful do you think you were in accomplishing the goals of the task set by the experimenter (or yourself)? How satisfied were you with your performance in accomplishing these goals?
Effort	Low/High	How hard did you have to work (mentally and physically) to accomplish your level of performance?
Frustration Level	Low/High	How insecure, discouraged, irritated, stressed, and annoyed versus secure, gratified, content, relaxed, and complacent did you feel during the task?

^1^The Performance subscale used by mistake the same endpoints as the others (the conventional endpoints are Good/Poor). The reported Performance values were, therefore, reversed during the analysis

To evaluate the subjective intrinsic motivation, participants responded to 31 questions (Table 6) selected from the well-established Intrinsic Motivation Inventory (IMI; Reynolds 2007). The full questionnaire consists of 45 questions and is divided into seven subscales. In the present study, only five subscales were selected: *Interest/Enjoyment* (7 questions), *Perceived Competence* (6 questions), *Effort/Importance* (5 questions), *Pressure/Tension* (5 questions), and *Relatedness* (8 questions). Participants responded on a Likert scale between 1 and 7 points; 1 indicated “Not at all”, 4 indicated “Somewhat true”, and 7 indicated “Very true”.

**Table 6:** Subscales and questions from the “Intrinsic Motivation Inventory” (IMI; Reynolds 2007). (R) indicates questions that were reversed when averaged in the analysis

Subscale	Question
Interest/Enjoyment	I enjoyed doing this activity very much
	This activity was fun to do
	I thought this was a boring activity (R)
	This activity did not hold my attention at all (R)
	I would describe this activity as very interesting
	I thought this activity was quite enjoyable
	While I was doing this activity, I was thinking about how much I enjoyed it
Perceived Competence	I think I am pretty good at this activity
	I think I did pretty well at this activity, compared to other students
	After working at this activity for a while, I felt pretty competent
	I am satisfied with my performance at this task
	I was pretty skilled at this activity
	This was an activity that I couldn’t do very well (R)
Effort/Importance	I put a lot of effort into this
	I didn’t try very hard to do well at this activity (R)
	I tried very hard on this activity
	It was important to me to do well at this task
	I didn’t put much energy into this (R)
Pressure/Tension	I did not feel nervous at all while doing this (R)
	I felt very tense while doing this activity
	I was very relaxed in doing these (R)
	I was anxious while working on this task
	I felt pressured while doing these
Relatedness	I felt really distant to this person (R)
	I really doubt that this person and I would ever be friends (R)
	I felt like I could really trust this person
	I’d like a chance to interact with this person more often
	I’d really prefer not to interact with this person in the future (R)
	I don’t feel like I could really trust this person (R)
	It is likely that this person and I could become friends if we interacted a lot
	I feel close to this person

The usability of the three different visualization technologies was evaluated with the “System Usability Scale” (SUS; Brooke 1996), widely employed for the usability assessment of software and hardware solutions (Faria et al. 2013; Meyer et al. 2019). The SUS measures several aspects of usability from *Effectiveness* (can the participant successfully achieve the task goals?) to *Efficiency* (how much effort is needed to perform the task?), and *Satisfaction*. The SUS consists of 10 questions (Table 7) with five response options on a Likert scale, from “Strongly agree” to “Strongly disagree”. Contrary to the conventional questionnaire, and due to an implementation lapse, a 7-point Likert scale (the same as the IMI) was employed instead of the standard 5-point scale.

**Table 7:** Questions from the “System Usability Scale” (SUS; Brooke 1996)

Question
I think that I would like to use this system frequently
I found the system unnecessarily complex
I thought the system was easy to use
I think that I would need the support of a technical person to be able to use this system
I found the various functions in this system were well integrated
I thought there was too much inconsistency in this system
I would imagine that most people would learn to use this system very quickly
I found the system very cumbersome to use
I felt very confident using the system
I needed to learn a lot of things before I could get going with this system

To assess embodiment, we selected questions from the established embodiment questionnaire (Kalckert and Ehrsson 2012; Longo et al. 2008). The questions were adapted based on the literature and tailored to our specific experiment to cover all three embodiment components, namely, *Body ownership*, *(Self-)location*, and *Agency* (Kilteni et al. 2012a; Longo et al. 2008). *Body ownership* describes the cognition that a body and/or limb is part of and belonging to the own body (Blanke 2012). *Location* refers to the knowledge of where one’s body and/or its parts are in space (Blanke 2012). Finally, *Agency* describes the experience that oneself is initiating and controlling an external event through one's own action (Braun et al. 2018; Haggard and Tsakiris 2009). The questions, their weight during analysis, and their targeted component of embodiment can be found in Table 8. Participants responded on a Likert scale between 1 and 7 points; 1 indicated “Not at all”, 4 indicated “Somewhat true”, and 7 indicated “Very true”.

**Table 8:** Components and questions from the Embodiment questionnaire (Q1–Q5: Longo et al. 2008; Q6: Kalckert and Ehrsson 2012) with the weightings used to compute the overall embodiment score

#	Component	Weight	Question
Q1	Body Ownership	1/3	It seemed like the virtual hand was my hand
Q2	Body Ownership	1/3	It seemed like the virtual hand was part of my body
Q3	Body Ownership	1/3	It seemed like I was looking directly at my own hand
Q4	Location	1	It seemed like my hand was in the location where the virtual hand was
Q5	Agency	1/2	It seemed like I was in control of the virtual hand
Q6	Agency	1/2	It seemed like I was causing the movements I saw

### Data analysis

A single value per questionnaire was computed following their specific conventions. The RTLX values from the 100 points slider scale were averaged across all subscales (Hart 2006). As the *Performance* subscale in RTLX (Table 5) used a wrong scale, we reversed it. The IMI values were averaged per subscale and the mean subscale values averaged across all subscales resulting in one inventory score (overall motivation; Reynolds 2007). The SUS values on the Likert scale were rescaled to a range from 0 to 100 and averaged across all items (Brooke 1996). Finally, the embodiment questions were also averaged, using the weightings listed in Table 8, so each component of embodiment (*Body ownership*, *Location*, and *Agency*) had the same influence on the overall embodiment value.

To investigate whether the subjective reports differentiate across modalities, a one-way repeated-measures analysis of variance (ANOVA) with the factors visualization modality (IVR, AR, 2D screen) was performed. Post-hoc pairwise comparisons with Tukey corrections were performed to compare levels of factors. The significance threshold was set at α < *0.05*. Data analyses were performed in Python 3.7.1 using the package *rpy2* version 2.9.4.

## Results

### Demographic

Based on EHI results, all participants had a positive lateral quotient (LQ) indicating preferences to use the right hand. More in-depth analysis based on other classification (Fagard et al. 2015), revealed that 16 participants where strongly right-handed (LQ <  =  + 90), three were mixed right-handed (+ 60 <  = LQ <  =  + 80), and one might be classified as ambidextrous (LQ =  + 20). Other descriptive statistics of the demographic information are reported in Table 9.

**Table 9:** Descriptive statistics of the demographic data

Measure	Scale	Average	Standard deviation	Min	Max
Handedness	[−100: 100]	91	19.71	20	100
EHI lateral quotient (LQ)	−100: strongly left-handed; 100: strongly right-handed				
I already have experience with Virtual Reality	7-point Likert scale 1: “not at all”; 4: “somewhat true”; 7: “very true”	2.4	1.76	1	7
I have experience with gaming	7-point Likert scale 1: “not at all”; 4: “somewhat true”; 7: “very true”	3.1	2.05	1	7
In the last month, I have spent an average of ____ hours per week gaming	Open field for numerical values (max: 168)	0.53	1.39	0	5

### Subjective reports

**Fig. 4:**
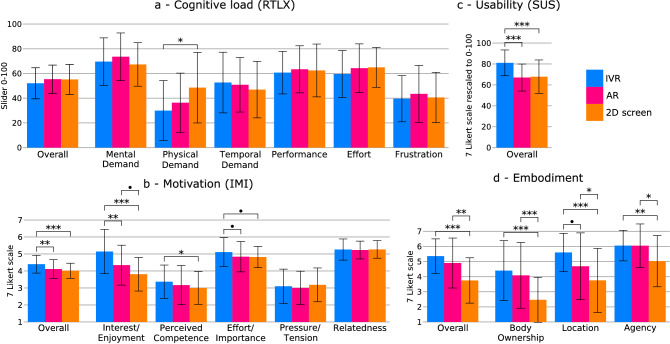
Mean scores reported after the immersive virtual reality (IVR), augmented reality (AR), and 2D screen modalities. **a** Cognitive load (RTLX) and its subscales; **b** Intrinsic motivation (IMI) and its subscales; **c** Technology usability (SUS); **d** Embodiment and its components. *** *p* < 0.001, ** *p* < 0.01, * *p* < 0.05, *p* < 0.1. Error bars: ± 1 SD

The results from the ANOVA on each questionnaire and subscale are listed in Table 10. We found a significant main effect of the visualization modality on the IMI, SUS, and embodiment scores (Fig. 4). The average value of the subjective cognitive load (RTLX) was lower with IVR compared to the other modalities, but differences did not reach significance (Fig. 4a). However, we found a significant main effect of the modality on the *Physical Demand* subscale of the RTLX. Regarding differences in the IMI subscales, we found a main effect of the visualization modality on the *Interest/Enjoyment*, *Perceived Competence*, and *Effort/Importance* subscales (Fig. 4b). We also found a main effect of modality in all the embodiment components (Fig. 4d).

**Table 10:** Main effect of the visualization modalities on self-reported cognitive load (RTLX), intrinsic motivation (IMI), technology usability (SUS), and embodiment

Questionnaire	df	F	Sig.
RTLX	2	1.14	0.32
RTLX: Mental demand	2	1.63	0.209
RTLX: Physical demand	2	4.45	0.021 *
RTLX: Temporal demand	2	0.94	0.401
RTLX: Performance	2	0.18	0.835
RTLX: Effort	2	0.91	0.404
RTLX: Frustration	2	0.01	0.985
IMI	2	13.64	< 0.001 ***
IMI: Interest/Enjoyment	2	17.19	< 0.001 ***
IMI: Perceived Competence	2	3.32	0.047 *
IMI: Effort/Importance	2	3.37	0.045 *
IMI: Pressure/Tension	2	0.46	0.636
IMI: Relatedness	2	0.05	0.951
SUS	2	11.78	< 0.001 ***
Embodiment	2	15.34	< 0.001 ***
Embodiment: Body ownership	2	13.89	< 0.001 ***
Embodiment: Location	2	10.82	< 0.001 ***
Embodiment: Agency	2	6.62	0.004 **

Bold highlighting indicates statistical significance

*** *p* < 0.001, ** *p* < 0.01, * *p* < 0.05$, **·** *p* < 0.1

Post-hoc comparisons are presented in Table 11. We found higher values in the **RTLX** subscale *Physical Demand* with the 2D screen than with IVR (Fig. 4a). We also found that, with IVR, participants reported higher **motivation** values than with AR and 2D screen (Fig. 4b). When investigating the IMI’s subscales, higher *Perceived Competence* and *Interest/Enjoyment* were reported with IVR compared to the 2D screen. The *Interest/Enjoyment* with IVR was also higher than AR, and AR showed a trend of higher *Interest/Enjoyment* compared to the 2D screen. Although the modality did impact the *Effort/Importance* significantly, the post-hoc analysis only resulted in a trend towards higher values with IVR compared to the other two modalities. We also found higher values in **usability** with IVR compared to AR and 2D screen (Fig. 4c). Finally, the **embodiment** was higher with IVR and AR compared to the 2D screen (Fig. 4d). Interestingly, no significant differences in embodiment were observed between IVR and AR. The three components of embodiment were, as the overall embodiment, significantly higher with the HMDs than with the 2D screen. The only difference between HMDs was the *Location* component that showed a trend for being lower with AR than IVR.

**Table 11:** Post-hoc paired t-tests

Item/scale	Group 1	Group 2	T	Effect Size	Sig.
RTLX: Physical demand	IVR	AR	− 1.008	0.45	0.577
	IVR	2D screen	− 2.936	0.83	0.015 *
	AR	2D screen	− 1.929	0.69	0.145
IMI: Overall	IVR	AR	3.705	0.88	0.002 **
	IVR	2D screen	5.040	0.93	< 0.001 ***
	AR	2D screen	1.335	0.56	0.385
IMI: Interest	IVR	AR	3.489	0.87	0.004 **
	IVR	2D screen	5.825	0.95	< 0.001 ***
	AR	2D screen	2.336	0.76	*0.063*
IMI: Competence	IVR	AR	1.404	0.57	0.349
	IVR	2D screen	2.575	0.79	0.037 *
	AR	2D screen	1.170	0.50	0.478
IMI: Effort	IVR	AR	2.165	0.73	*0.090*
	IVR	2D screen	2.325	0.76	*0.064* **·**
	AR	2D screen	0.160	0.08	0.986
SUS	IVR	AR	4.326	0.91	< 0.001 ***
	IVR	2D screen	4.069	0.90	< 0.001 ***
	AR	2D screen	-0.257	0.13	0.964
Embodiment	IVR	AR	1.518	0.60	0.294
	IVR	2D screen	5.373	0.94	< 0.001 ***
	AR	2D screen	3.855	0.89	0.001 **
Embodiment: Body Ownership	IVR	AR	0.805	0.37	0.702
	IVR	2D screen	4.913	0.93	< 0.001 ***
	AR	2D screen	4.108	0.90	< 0.001 ***
Embodiment: Location	IVR	AR	2.202	0.74	*0.084*
	IVR	2D screen	4.649	0.92	< 0.001 ***
	AR	2D screen	2.447	0.77	0.050 *
Embodiment: Agency	IVR	AR	0.089	0.04	0.996
	IVR	2D screen	3.194	0.85	0.008 **
	AR	2D screen	3.106	0.84	0.010 *

Bold highlighting indicates statistical significance

*** *p* < 0.001, ** *p* < 0.01, * *p* < 0.05$, **·** *p* < 0.1

## Discussion

We investigated the impact of three visualization technologies—HTC Vive HMD for more immersive VR (**IVR**), Meta 2 HMD for augmented reality (**AR**), and 2D computer **screen**—on subjective reporting of cognitive load, intrinsic motivation, technology usability, and embodiment of a virtual hand in a dual motor-cognitive task (i.e., reaching fruits and counting different fruit types) with 20 healthy young participants. The currently presented results complement our previous analysis on the movement quality and cognitive load measured through the cognitive counting task (Wenk et al. 2019).

### The visualization modality did not impact the subjectively reported cognitive load

Previous research has hypothesized that differences in motor performance across visualization technologies might derive from an increase in the cognitive load resulting from the visuospatial transformation between the real movement and its visualization (Mousavi Hondori et al. 2016). In our previous analysis, we found that IVR enhanced motor performance when compared to the 2D screen display (Wenk et al. 2019). However, we did not observe differences in the cognitive load objectively measured through the parallel cognitive counting task. We hypothesized that participants might have prioritized success on the cognitive task over the reaching task. If such a prioritization took place, it might still be possible that participants subjectively perceived differences in their cognitive load. Therefore, we evaluated participants’ self-reported cognitive load measured through the “Raw Task Load Index” (RTLX; Hart 2006) questionnaire. The presented study is, to the best of our knowledge, the first attempt to measure differences in subjectively reported cognitive load across visualization technologies in a 3D reaching task.

Contrary to our expectations, but in line with our previous analysis on the cognitive counting task, no significant differences were found in the overall self-reported cognitive load across modalities, challenging the hypothesis that participants prioritized the cognitive task over the motor one. Importantly, the *Physical Demand* subscale of the RTLX reached statistical significance. In particular, the reported physical demand was significantly higher in the 2D screen modality, compared to IVR. As reported in (Wenk et al. 2019), participants performed less straight and smooth movements with the 2D screen visualization, especially when reaching targets that required movements in more than one dimension. Therefore, a possible rationale behind the differences in physical demand might be the reduced and unnatural depth visualization with the 2D screen. Further, in the 2D screen modality, participants did not look at their hands but at the 2D screen, disrupting the eye-hand coordination and, likely, increasing the physical demand.

We did not find significant differences between the optical see-through AR system and the 2D screen in any RTLX subscales. This is not in line with a study that compared different AR visualization technologies to a 2D computer screen (Baumeister et al. 2017). Authors compared the effect of three AR display technologies—spatial AR (a projection system), the optical see-through (OST) HMD Microsoft HoloLens, and the video see-through (VST) HMD Samsung Gear VR—on healthy participants’ cognitive load and performance during a button-pressing task. Authors reported a lower cognitive load—measured with a dual-task and a self-reporting questionnaire (Paas scale; Paas 1992)—and better motor performance (i.e., faster response time) with the spatial AR compared to the 2D screen. However, better motor performance and lower cognitive load were observed in the 2D screen vs. the AR HMDs. With HMDs, as with the projection, the movements are usually visualized in the same space where they are performed, removing the demanding visuospatial transformations. However, some transformations remain with HMDs (e.g., a perspective shift with VST AR HMDs due to the misalignment between the cameras and the eyes) that together with other limiting aspects (e.g., reduced depth cues, reduced field of view, incapacity to render opaque images with OST AR, etc.) might impact the movements’ perception. We note, however, that (Baumeister et al. 2017) focused on the cognitive load associated with receiving instructions for a button-pressing procedural task (i.e., a decision-making task), while we evaluated the cognitive load associated with reaching movements in the 3D space.

Our experiment, as most of the previous studies that evaluated cognitive load during different visualization modalities, was performed with young healthy subjects. To date, little has been shown on the effect of different displays in an older population, despite its relevance in neurorehabilitation (Huygelier et al. 2019). Neurologic injuries, such as stroke, are more prevalent at older ages. Furthermore, cognitive changes, sensory and motor limitations, and slower speed of processing in the very old population (Cabeza 2001; Park and Reuter-Lorenz 2009) are some characteristics associated with participants’ age that might influence their cognitive load. Thereby, studying the visualization technology that could decrease the cognitive load with healthy old participants might draw different results than the ones observed with a healthy young population. Further studies with healthy elderly and neurologic patients would help to get a better insight into VR-based neurorehabilitation.

### Immersive virtual reality promotes participants’ motivation

We hypothesized that visualizing the task with both IVR and AR HMDs would result in higher self-reported levels of motivation than with the 2D screen. Results from the IMI questionnaire partially support our hypothesis. The participants’ reported motivation was higher with IVR than with the 2D screen. Higher reported values for IVR are especially visible in the *Interest/Enjoyment* subscale—the subscale directly assessing intrinsic motivation—where the difference is not only statistically significant but also more pronounced than in other subscales. The higher levels of self-reported motivation and enjoyment might be due, in part, to the skillful movements accomplished with IVR (i.e., faster, smoother, and straighter movements). Task difficulty and motivation are closely related and have been extensively researched (Ach 1935). The significantly higher levels of self-reported *Perceived Competence* observed in the IVR visualization supports this interpretation. This is in line with previous studies that found IVR HMDs to enhance participants’ motivation, compared to 2D computer screens, in an electric-powered wheelchair simulator (Rivera-Flor et al. 2019), and a full-body movement task (Born et al. 2019). Importantly for neurorehabilitation, higher levels of participants’ intrinsic motivation and perceived competence during motor training have been shown to improve the consolidation of new motor skills (Trempe et al. 2012; Widmer et al. 2016). Therefore, IVR HMDs have a great potential to enhance neuroplasticity underlying motor recovery.

Contrary to our expectations, the AR visualization did not result in higher levels of self-reported motivation when compared to the 2D screen. Although the level of *Interest/Enjoyment* was higher with AR than the 2D screen, differences did not reach significance. Importantly, we observed a significantly lower motivation level during AR compared to IVR, especially in the *Interest/Enjoyment* subscale. The reason behind the relatively low motivation levels associated with AR is unclear. In our previous motor performance analysis, we observed a better movement quality with AR vs. 2D screen but worse than IVR. However, the differences did not reach significance. The reported *Perceived Competence* reflects those findings—i.e., the perceived competence with AR was higher than with 2D screen, but lower than IVR, without reaching significance. Therefore, the significantly lower intrinsic motivation associated with the AR visualization does not seem to be exclusively linked to a lower perceived competence. The technical limitations of the AR HMD might have played a crucial role in participants’ motivation—e.g., the AR HMD was the display with the smallest field of view (see Table 4) and a small spatiotemporal shift from the real to the virtual controller was noticeable and, probably, uncomfortable (Wenk et al. 2019).

Although several studies have integrated HMDs to increase motivation (Dias et al. 2019; Gamito et al. 2014), they did not systematically measure participants’ motivation. Only a few of the studies using games for motivation purposes in rehabilitation evaluated this important factor (Swanson and Whittinghill 2015). Although the overall motivation score in our experiment is rather low (just above “somewhat true”), we note that our study aimed to compare the visualization modalities rather than to increase the overall motivation—i.e., no gamification elements were included. Moreover, the parallel counting task was designed to be almost impossible to be accomplished without mistakes, as we relied on the number of failures to measure the cognitive load (Wenk et al. 2019). This might be the main reason behind the relatively low levels of perceived competence across modalities.

### Immersive virtual reality was reported as the most usable visualization

As hypothesized, we found higher levels of usability in the IVR HMD visualization—evaluated with the “System Usability Scale” (SUS; Brooke 1996)—compared to the 2D screen. Our results on higher usability but equal cognitive load for the IVR vs. the 2D screen are in line with studies suggesting that usability and mental workload are two non-overlapping concepts that should be addressed independently, but that, when jointly enhanced, might significantly improve human performance (Longo 2018). High usability has been consistently reported alongside high motivation for games or VR applications in the fields of cognitive rehabilitation (Mondellini et al. 2018; Rocha et al. 2016; Sokolov et al. 2020), motor rehabilitation (Nijenhuis et al. 2015; Radder et al. 2016), and self-awareness (Llorens et al. 2015). A correlation between motivation and usability in online learning platforms has also been reported (Hu 2008). This correlation might help to interpret our findings. In line with previous studies, the higher usability scores for the IVR (reflected in SUS) might be related to the reported higher *Interest/Enjoyment* in the IMI for this modality.

Contrary to our expectation, participants reported lower levels of usability during the AR modality compared to IVR. The AR modality might have been impacted negatively by the technical limitations of the state-of-the-art display employed in this study—e.g., by focal rivalry. Introduced by (Oshima et al. 2016), focal rivalry designates the continuous accommodation that the eye needs to perform when switching between real and virtual elements, as the focal distances with the current off-the-shelves AR HMDs are fixed. Focal rivalry prevents real and virtual objects that are at the same distance (based on the eye’s vergence) from being seen sharp at the same time (unless the focal distance of the real object corresponds, by chance, to the HMD focal distance). The Meta 2 HMD is not equipped with lenses, resulting in a reflected image on the transparent glass with a focal distance inferior to 20 cm. Since no fruit location was closer than 30 cm to the participants’ eyes, we can assure that this focal rivalry was perceptible by the participants. The focal rivalry has been found to deteriorate motor performance in a high-precision manual task guided by virtual elements (Condino et al. 2020). Participants performed significantly worse when drawing lines between real and virtual dots rendered with an OST AR HMD than when connecting the dots without AR. However, and in line with our results, Condino et al. did not report an effect of focal rivalry on the cognitive load (assessed with the NASA-TLX; Hart and Staveland 1988). Since eye accommodation and vergence are predominately unconscious reflex actions to maintain focus on an object, the focal rivalry conflict might result in a general (physical) discomfort related to visual fatigue that participants do not associate with (cognitive) task demands. Indeed, participants in (Condino et al. 2020) seem not to be aware of the consequences of focal rivalry on motor performance. Therefore, potential discomfort associated with focal rivalry may be reflected in decreased usability ratings rather than enhanced cognitive load. Moreover, other technical limitations, such as the setup time (i.e., the calibration time was the longest among all the devices: ~ up to 5 min), the incapacity to render opaque images with OST HMDs, and the small field of view (the AR HMD had the smallest field of view among all the displays) might limit the potential incorporation of AR HMDs into regular clinical use.

The fact that one of the two novel HMD devices, which were similarly hypothesized to show advantages over the 2D screen, was rated more useable than the other highlights the importance of assessing system usability when comparing technologies. These results might reflect the actual infant state of the technology. However, most of the identified limitations—e.g., the restricted field of view or focal rivalry—might be overcome with new hardware development in the near future (Zhan et al. 2020). Nevertheless, it is important to test the usability of the new HMD technology, both with therapists and patients, to reduce the risk to prematurely apply technology that is in its infancy but currently making rapid strides in development.

### HMDs enhance embodiment

We found that, as expected, the sense of the embodiment over the virtual hand was significantly higher with both HMDs compared to the 2D screen. Significant differences were observed in all the embodiment components (i.e., *Body ownership*, *Location*, and *Agency*). This is in line with a recent study that found higher embodiment values with an IVR HMD compared to a 2D computer screen in a VR task requiring hand movements in the 3D space (Juliano et al. 2020). Our study consolidates this finding and shows similar values, especially regarding the *Location* component, supporting our hypothesis on a more realistic immersion in the VE provided by the HMDs than the 2D screen.

However, AR did not result in a higher embodiment level compared to IVR. When having a closer look at the three components of embodiment, we found a higher *Location* level with IVR, compared to AR, that approached statistical significance. The absence of higher embodiment with AR might be due to the wording employed in the questionnaire. Two over three body ownership questions (Q1 and Q2), the location question (Q4), and half of the agency questions (Q5) were referring to a virtual hand that was not present in the AR modality. Those questions might have been, therefore, unclear. Although the questions might have been less ambiguous in the 2D screen modality, the fact that both the real and the virtual hand were visible at the same time, might explain the high variance observed in the 2D screen responses, especially in the *Location* component. Alternatively, the low *Location* values reported with AR might also be explained by some of the technical limitations faced with AR—e.g., a small spatiotemporal shift visible between the real and virtual controller (with the virtual blue ball) during fast movements (Wenk et al. 2019).

The observed high general embodiment values with IVR are in line with recent studies that compared HMDs to 2D screens. In the study by Borrego et al., the authors found similar embodiment levels in healthy participants in the more immersive VR modality (Borrego et al. 2019). They also included a 2D screen modality, but the 2D screen displayed the avatar at a third-person perspective, making it less comparable to our first-person perspective approach. In (Born et al. 2019), authors compared an IVR HMD to a 2D computer screen with a first- or third-person perspective. The IVR HMD led to higher embodiment values for each of its components. Further, the first-person perspective led to higher body ownership and location values compared to the third-person perspective. However, their study design did not allow to compare the first-person perspective in HMD to the one in the 2D screen.

The embodiment component that reached the highest values across all visualization modalities was *Agency*. Although the animation of the virtual participants’ arm was not exactly the same as the participants’ arm—i.e., we used inverse kinematics (IK) to animate the avatar’s arm using the position and orientation of the controller—the level of agency with IVR still reached a mean value of 6.06 ± 1.01 (over 7). The real hand’s position and orientation were, therefore, respected in the VE, but the more proximal arm segments (i.e., upper and lower arm) were not. However, the reaching task did not require attention to the whole arm. Furthermore, our questionnaire did not measure the whole arm or body embodiment, but rather the embodiment towards the (virtual) hand—i.e., all the questions except the last one (over six) asked specifically about the (virtual) hand. The lack of negative impact due to IK on embodiment was also found in a recent study that compared an IK implementation versus a motion capture system for computing forward kinematics (FK) animations (Parger et al. 2018). The authors found high embodiment levels in IK, even higher than FK for some tasks, as the motion capture system was not always reliable. It seems reasonable to think that the high embodiment values observed with IVR (especially in *Agency*) reflect a good acceptance of the IK animation in our reaching task. The lower agency reported in the 2D screen modality might result from the lower capacity of this display to render the depth, reducing participants’ awareness that their action along the z-axis had an impact in the VR.

*Body ownership* was the component with the smallest reported values across displays. As realism appears to impact body ownership (Argelaguet et al. 2016), the observed lower body ownership level might be due to the avatar’s appearance. The animated avatar did not look exactly like the participants’ arms (e.g., the avatar always wore a blue long-sleeve shirt) and participants might not have held the controller with the same cylindrical grip as the avatar did. A large body of research has shown that body ownership illusions can be experimentally induced in a part of a body or an entire body other than one's own (e.g., fake physical or virtual hand). For the illusion to work, it is usually required that the fake hand looks like a hand and that it is aligned with the orientation of the real hand (e.g., Ehrsson et al. 2004; Haans et al. 2008; Pavani et al. 2000; Tsakiris and Haggard 2005).

However, the brain also seems to be tolerant to some extent when it comes to accepting a virtual limb as an own limb. For example, studies have successfully induced body ownership over elongated arms (Kilteni et al. 2012b), heavier bodies (Normand et al. 2011), and mismatched skin-colored arms (Banakou et al. 2016; Peck et al. 2013). Importantly, adding visuomotor synchronies—i.e., movements are performed with the avatar instead of having a static avatar—have shown to induce strong embodiment, even if the virtual limb’s appearance is very different from the (own) human body (Banakou et al. 2016; Normand et al. 2011; Peck et al. 2013). This could explain why, in our study, we found high overall embodiment levels using our HMDs, more pronounced in the agency than in the body ownership component.

Although several studies have used HMDs to show beneficial effects of virtual embodiment on motor task performance (Grechuta et al. 2017; Odermatt et al. 2021; Shibuya et al. 2018), they did not primarily aim at studying how different visualization technologies affect embodiment levels. Our results complement these studies and suggest that AR HMD, which allows patients to see their own arm/limb, may not necessarily support embodiment. More crucially, a consistent virtual environment, as provided with IVR HMD, may be sufficient to support embodiment, even with a relatively easy-to-implement movement visualization using IK algorithms and generic humanoid avatars. Therefore, IVR HMDs are a promising tool to design more efficient motor training paradigms in neurorehabilitation.

### Implications for stroke neurorehabilitation

The results from this study provide relevant information to improve movement training in unimpaired users using more immersive displays. Furthermore, our findings are also relevant to the neurorehabilitation field. In VR-based therapy, task-specific functional 3D movements are visualized in virtual environments that offer the possibility to simulate different real or imaginary activities of daily living that can be adapted to the patients’ needs, while providing a motivating (Lee et al. 2003) and safe environment (Marchal-Crespo and Reinkensmeyer 2008). During conventional VR-based neurorehabilitation, the VE is displayed on a 2D screen and patients interact via a symbolic virtual representation of their limb (e.g., a cursor). Although this provides useful visual guidance (Basalp et al. 2019; Marchal-Crespo et al. 2019, 2013), the lack of some depth cues (i.e., stereopsis and motion parallax) requires movements and object interactions that are far from those required in real conditions, limiting patients’ opportunity to embody the virtual avatar and transfer of acquired skills to real practice (Bezerra et al. 2018; de Mello Monteiro et al. 2014). Our work is the first step to surpass these shortcomings as it provides a better understanding of the effects of different visualization technologies on subjective measures of cognitive load, motivation, technology usability, and embodiment that might have an important impact on neurorehabilitation.

### Future research

Performing a similar study with neurologic patients, especially those suffering from cognitive impairments, is our next step. Experiments with brain-injured patients might reveal different results from the ones here presented, especially on the reported cognitive load. Questionnaires measuring the self-reported cognitive load, such as the NASA Task Load Index might be unsuited in a brain-injured population as they might lack the full awareness of their cognitive limitation (Cyr et al. 2009). As stroke patients might suffer from sensorimotor deficits that prevent them from performing unsupported reaching movements, we need to adapt our experimental setup to interface the developed VR reaching task with an upper-arm assisting rehabilitation device (e.g., Marchal-Crespo et al. 2017; Özen et al. 2020). A study with patients might also result in higher variance in our measurements due to the wide range of potential motor and cognitive disabilities. Neurological injuries are more prevalent at older ages and older adults are less technology experienced. Thus, stroke patients might not be willing to be immersed in VE using HMDs. However, based on our own experience (Gerber et al. 2017) and recent research with older adults (Huygelier et al. 2019) and stroke patients (Borrego et al. 2019), we are confident that stroke patients’ attitudes towards a more immersive VR would be positive and would embody the virtual avatar.

A further important point to be addressed in future studies is to investigate how the technical limitations of a given VR system could affect users’ performance. The user’s experience assessed with subjective reports, which depict the primary focus of this work, is only one of several important components that have to be taken into account to test the feasibility of VR systems for clinical applications. Knowing how the system’s technical limitations could affect the movement precision in tasks commonly trained in therapy is important before selecting a technology. Performing VR benchmarks assessing the required task performance (e.g., maximum movement precision to expect) and limitations of a given VR system/application may, therefore, be a promising and important approach to evaluate the feasibility of clinical VR systems for potential applications in neurorehabilitation (Otto et al. 2019).

Finally, if ongoing studies continue to indicate that IVR is the best-suited visualization technology, future work may emphasize on how to optimize the benefits from the use of this new technology to design more meaningful future VR-based interventions. For example, IVR offers the potential to shift the patients’ presence from a clinical environment to a fully controllable VE, allowing control over what patients visually perceive during therapy. Future research may systematically investigate how different elements in the VE, for example, used as visual distractors, impact patients’ motor and cognitive performance. Further, considering the fact that attentional disorders are highly prevalent in stroke patients (Patel and Birns 2015), controlling the amount of elements or distractors may be a powerful tool to adapt therapies to the patient’s need or provide patient-tailored attentional training. The adaptability of the VE in IVR could further allow to immerse patients into familiar or pleasant environments, potentially enhancing their engagement and motivation during the training.

### Study limitations

The subjective reports in the AR modality resulted in intermediate values between IVR and the 2D screen, preventing clear conclusions on the effectiveness of this novel modality. Aside from the small sample size (20 participants) that might have prevented us from reaching statistical significance, three confounds might have influenced our AR results. First, a small spatiotemporal shift was visible between the real controller and the virtual blue ball rendered on the controller. This shift, which was especially visible in fast movements, might have affected the reported values of usability, motivation, and the embodiment’s component *Location*. Second, the questions from the embodiment questionnaire might have been misleading since some were referring to a virtual hand not present in the AR modality. Finally, and probably the most decisive factor, the employed AR display (Meta 2, Meta View, USA) suffered from several technical limitations. The perceptual limitation typically observed in optical see-through devices, such as focal rivalry (Condino et al. 2020), might have diminished the potential benefits of a more direct visuospatial transformation or the vision of the participant’s own body parts on the cognitive load, motivation, technology usability, and embodiment.

Some implementation lapses in the questionnaires’ endpoints might have influenced some subscales results. The *Performance* subscale of the RTLX (Table 5) had non-conventional endpoints (i.e., Low/High vs. Good/Poor). We, therefore, reversed the participants’ answers in this subscale to better interpret the results. We also used the SUS with non-conventional endpoints—i.e., a 7-point Likert scale (the same as the IMI) was employed instead of the standard 5-point scale. However, we do not expect that this more sensitive point scale alters our results. Finally, the randomization order of the three visualization modalities resulted in 45% of the participants starting with IVR and 25% with the 2D screen. This unbalanced order perhaps influenced the motivation, usability, and embodiment in IVR compared to the 2D screen. Participants might have been more fatigued and unmotivated towards the end of the experiment (even though the dual-task took only 5 min per modality). However, they might have also learned the location of the fruits in the previous tests, resulting in better performance during the last test.

Further, our results might be specific to the task required in our study—i.e., reaching to non-tangible objects in the 3D space—and generalization to other tasks should be taken cautiously. We decided to employ a rather simple task to limit the possible differences in self-reported metrics between visualization technologies to be only due to the change in movement visualization itself. For example, for all display types, the task was the same: moving the right arm to reach virtual fruits with an HTC Vive’s controller held in the hand. The participants did not press any buttons or perform extra actions (e.g., grab, throw, validate, etc.) and did not have to orient the controller in specific rotation to manipulate objects (e.g., rotating a piece in an assembly task). The goal of this rather simplistic interaction mode was to enhance the system’s flexibility and facilitate the interface between our system and any mechanical rehabilitation devices currently located in the clinics and likely increase patients’ and therapists’ acceptance. Of equal importance, the simple interaction mode would also be compatible with many rehabilitation interventions—i.e., performing movements with the affected arm. Future studies are needed to compare and test how visualization technologies affect different tasks (e.g., bimanual assembly task, grasping, etc.) and task performance of VR exercises with different interaction modes (e.g., button pressing/holding, bare-hand interaction, bimanual object manipulation, etc.).

A further important aspect to consider when interpreting our results is the realism and complexity of the VE. Even though the VEs were composed of the same virtual elements for the 2D screen and IVR, participants could visually explore the VE by moving their heads while wearing the HMD. A larger portion of the VE was then visible in IVR compared to the fixed-perspective displayed on the 2D screen. The VE aimed to reproduce the uncluttered experimental room (i.e., table, walls, lights). However, the physical environment was still richer than the VE as it contained the researcher, some hardware and cables, and the real participant’s body instead of an avatar. Importantly, more complex environments were found to worsen the performance in a visual scanning task (time to find a target) (Ragan et al. 2015). Therefore, performing the same task in IVR with a low-complexity VE might have led to better performance than the more realistic complex environment perceived with AR (Lee et al. 2013). Although our task was designed to reduce visual exploration by selecting a task workspace that fits in the participants’ field of view, it is still possible that the more complex physical environment (visible in AR) disrupted the participant’s ability to detect the fruits. This could have been reflected in a more frustrating task (lower motivation), and/or a system perceived as less usable. Therefore, before generalizing our results, it would be worth investigating if increasing the realism and complexity of the VE to better match the physical environment seen with AR would change our findings.

## Conclusions

This paper presents results from the evaluation of the impact of different visualization displays (an HTC Vive HMD for IVR, a Meta 2 HMD for AR, and a 2D computer screen) on self-reported cognitive load, motivation, technology usability, and embodiment in healthy young participants. Participants performed a motor task that included reaching movements in the 3D space and a parallel counting task. No differences in the cognitive load across displays were observed. The participants’ self-reported intrinsic motivation and technology usability were higher with IVR than with AR and the 2D screen. Further, a higher embodiment level was reported with the two HMDs when compared to the 2D screen. Contrary to our expectation, the beneficial aspect of HMD was mostly observed with IVR, but not with AR, suggesting that the current state-of-the-art on augmented reality is not mature enough to be employed in neurorehabilitation settings. A more immersive VR employing HMD offers naturalistic interactions in engaging training environments and has a great potential to not only make neurorehabilitation interventions more efficient, but also more accessible and adhering to patients.

## Data Availability

Raw data were generated at the Motor Learning and Neurorehabilitation Laboratory, ARTORG Center for Biomedical Engineering Research, University of Bern, Bern, SWITZERLAND. Derived data supporting the findings of this study are available from the corresponding author on request.

## Code availability

The source code will be made available in a later stage when ongoing experiments with elderly healthy participants and brain-injured patients are published.
